# *TET3‐*mediated demethylation in tomato activates expression of a *CETS* gene that stimulates vegetative growth

**DOI:** 10.1002/pld3.22

**Published:** 2017-10-30

**Authors:** Elizabeth Hollwey, Suzan Out, Michael R. Watson, Iris Heidmann, Peter Meyer

**Affiliations:** ^1^ Centre for Plant Sciences University of Leeds Leeds UK; ^2^ ENZA ZADEN Research and Development Enkhuizen The Netherlands

**Keywords:** CETS gene, DNA demethylation, DNA methylation, floral repressor, PEBP protein, *Solanum lycopersicum*, Ten‐Eleven‐Translocation (TET) proteins

## Abstract

Expression of the mammalian DNA demethylase enzyme *TET3* in plants can be used to induce hypomethylation of DNA. In tomato lines that express a *TET3* transgene, we observed distinct phenotypes including an increase in the length and number of leaves of primary shoots. As these changes resemble phenotypes observed in plants with strong expression of *SELF PRUNING (SP),* a member of the *PEBP/CETS* family, we investigated in *TET3* lines the expression levels of members of the *PEBP/CETS* gene family, which affect shoot architecture and growth of sympodial units in tomato. We did not detect any changes in *SP* expression in *TET3* lines, but for *CEN1.1*, a putative family member that has not been functionally characterized, we identified changes in gene expression that corresponded to hypomethylation in the upstream region. In tomato wild type, *CEN1.1* is expressed in roots, petals, and shoot apices but not in mature leaves. In contrast, in *TET3* transformants, the *CEN1.1* gene became hypomethylated and activated in leaves. Ectopic expression of *CEN1.1* in tomato caused similar phenotypes to those seen in *TET3* transformants. Vegetative growth was increased, resulting both in a delay in inflorescence development and in an instability of the inflorescences, which frequently reverted to a vegetative state. Ectopic expression of *CEN1.1* in *Arabidopsis thaliana* also caused floral repression. Our data suggest that the phenotypes observed in *TET3* lines are a consequence of ectopic activation of *CEN1.1,* which promotes vegetative growth, and that *CEN1.1* expression is sensitive to DNA methylation changes.

## 1 INTRODUCTION

Organization of shoot architecture in flowering plants is extremely important for the normal development of the plant, both under usual environmental conditions and when the plant is under stress. For crop plants such as tomato, shoot architecture also has great economic importance, with different patterns being preferred for different purposes. For example, for mechanically harvested processing tomatoes, tomato plants with determinate growth have a higher yield, while tomato varieties that grow indeterminately are better suited to produce tomatoes that are eaten fresh and require continuous market delivery (Jiang et al., 2013). Tomato is an example of a plant species with a sympodial growth pattern, composed of a series of determinate meristems. The primary shoot of tomato terminates with an inflorescence after 8–12 compound leaves (McGarry & Ayre, 2012), but growth continues from the uppermost axillary meristem (Lifschitz et al., 2006). After this point, the shoot is formed from repeating sympodial units consisting of three leaves and terminating with an inflorescence. Upward growth of the shoot is again continued from the most proximal axillary bud of the previous sympodial unit in an indeterminate fashion (Lifschitz et al., 2006).

The establishment of this pattern relies on the balance between the expression levels of genes in the tomato *PEBP* gene family (phosphatidylethanolamine‐binding protein), also called the *CETS* (*CENTRORADIALIS/TERMINAL FLOWER 1/SELF PRUNING*) gene family after its founding members (Shalit et al., 2009). This family is present in a large variety of species where it plays a role in mechanisms as diverse as bulb induction in onions and formation of needles in Norway spruce (Karlgren, Gyllenstrand, Clapham, & Lagercrantz, 2013; Lee, Baldwin, Kenel, McCallum, & Macknight, 2013; Wickland & Hanzawa, 2015). *SFT* (*SINGLE FLOWER TRUSS*), the tomato homolog of the *Arabidopsis thaliana* gene *FT* (*FLOWERING LOCUS T*; Lifschitz et al., 2006)*,* and *SP* (*SELF PRUNING*), the tomato homolog of the Arabidopsis gene *TFL1* (*TERMINAL FLOWER 1*; Pnueli et al., 1998)*,* are the best described of the genes in this family in tomato. Mutations in *SFT* result in delayed flowering (Lifschitz et al., 2006) while *sp* tomato mutants initially flower after the normal number of leaves has been produced but afterward flowers switch to determinate growth (Shalit et al., 2009). Overexpression of the *SFT* gene causes early flowering, the opposite phenotype to overexpressing *SP*, which results in delayed termination of the primary shoot and increased numbers of leaves per sympodial unit (Lifschitz et al., 2006; McGarry & Ayre, 2012; Pnueli et al., 1998). Analysis of double mutants indicates that *SP* counteracts the florigenic effect of *SFT* in a dosage‐responsive manner (Molinero‐Rosales, Latorre, Jamilena, & Lozano, 2003; Shalit et al., 2009). In addition to *SP* and *SFT*, there are several other recognized members of the *CETS* gene family in tomato (Cao et al., 2016; Carmel‐Goren, Liu, Lifschitz, & Zamir, 2003). Three of these (*SP5G*,* SP5G2,* and *SP5G3*) have been shown to have a role in delaying flowering, with knockdown lines of these genes showing early flowering and overexpression in Arabidopsis causing delayed flowering (Cao et al., 2016; Chitwood et al., 2013). Expression of *SP5G*,* SP5G2,* and *SP5G3* is affected by day length (Cao et al., 2016). Understanding the role of the genes in this family is an important tool to improve tomato crop yield or harvest index (yield per plant weight; Park et al., 2014; Soyk et al., 2017).

The likelihood of gene expression is frequently affected by epigenetic modifications to the gene, such as histone modifications and DNA methylation (Zilberman, Gehring, Tran, Ballinger, & Henikoff, 2007). DNA methylation occurs through the action of DNA methyltransferases and the presence of DNA methylation in the promoter of a gene is usually repressive, resulting in the silencing of that gene. Given the importance of tomato as a crop plant and the involvement of methylation in the ripening process of tomato (Liu et al., 2015; Zhong et al., 2013), a better understanding of the role of methylation in tomato is extremely important. Expression of the catalytic domain of the mammalian DNA demethylase *TET3 (TET3c)* in Arabidopsis has previously been shown to be capable of causing DNA demethylation (Hollwey, Watson, & Meyer, 2016).

Here, by transforming the *TET3c* construct into tomato, we observed specific phenotypes and demonstrated that expression of *CEN1.1,* a member of the *CETS* gene family, is affected by DNA methylation upstream of the start codon. We show that hypomethylation caused by *TET3c* results in the activation of this *CETS* family member. We demonstrate that ectopic expression of either *TET3c* or *CEN1.1* causes common phenotypes in tomato plants, including an instability of the transition to an inflorescence, delayed growth, and an increase in the number of leaves between inflorescences. Ectopic expression of *CEN1.1* in *Arabidopsis thaliana* also results in an increase in the number of rosette leaves and a delay in flowering.

## 2 MATERIALS AND METHODS

### 2.1 Vector construction and plant transformation

The *TET3c* vector was constructed as described in Hollwey et al. (2016). The *CEN1.1* vector was constructed by amplification of the *CEN1.1* genomic region from tomato DNA using primers GGGAAGCTTGGCACGTTGATTGGTTTTTCG + GGGAATTCACAAGCAAATGAGTAGGACAAACA. It was then cloned into the *HindIII/EcoRI* site of pGreen II 0029. The vectors were transferred into *Agrobacterium tumefaciens* for leaf disk transformation (Rai et al., 2012) of a EZCBT1 tomato variety and floral dip transformation of *Arabidopsis thaliana* (Col‐0; Clough & Bent, 1998). Tomato transformation was carried out at the premises of ENZA ZADEN, Enkhuizen, The Netherlands.

### 2.2 Plant material

Plants were grown in a growth chamber under long day conditions (16 hr light, 8 hr dark, 23°C, 42% humidity). At the age of 5 weeks, tomato plants were transferred to a glasshouse. All samplings for nucleic acid extractions were done between 8 and 10 a.m. to avoid possible circadian variations in gene expression or DNA methylation.

### 2.3 Expression analyses

RNA for expression analysis was extracted as described in Stam et al. (2000). DNA was removed using the TURBO DNase kit (Ambion applied Biosystems) and converted to cDNA using M‐MLV reverse transcriptase and oligo‐dT primers (Invitrogen) according to the manufacturer's instructions. Semiquantitative PCR was carried out using MyTaq Red DNA Polymerase (Bioline) and qPCR was carried out using SsoFast Eva Green Supermix (Bio‐Rad) according to the manufacturer's instructions. cDNA levels were normalized using eukaryotic translation initiation factor 3 primers GAGCGATGGATGGTGAATCT + TTGTACGTGCGTCCAGAAAG.

*CEN1.1* expression was analyzed using primers GACCCTGATGCTCCAAGTCC + TGGCTGCAGTTTCTCTCTGG.

### 2.4 DNA methylation analysis

Genomic DNA for bisulfite sequencing was extracted according to Vejlupkova and Fowler (2003) with some modifications. Tissue for the SAP methylation analysis was isolated using a dissection microscope from FFPE sections of tomato shoot apices made according to Vitha, Baluška, Jasik, Volkmann, and Barlow (2000). Bisulfite treatment was carried out using the EZ DNA Methylation‐Lightning kit (Zymo Research). Bisulfite‐treated DNA was amplified using primers AAYTTTTGGGGTGTGAGTTAGA + TCCACCCATTTCATTAACCACC and GTGAGGTGGGGTGTTAAAGAATGA + CACCRATRTAACACTCCACCT to amplify part of the region upstream of the *CEN1.1* gene. Oxidative bisulfite sequencing was performed as described in (Booth et al., 2013) to quantify levels of 5‐methylcytosine and subtracted from bisulfite sequencing data (which contains 5‐methylcytosine and 5‐hydroxymethylcytosine) to calculate levels of 5‐hydroxymethylcytosine; 10–20 clones were sequenced per sample. Sequencing data were analyzed using the online CYMATE tool (Hetzl, Foerster, Raidl, & Scheid, 2007) and the program SequenceFileConverter (J. Royle).

## 3 RESULTS

### 3.1 *TET3c* tomato plants display abnormal growth phenotypes and ectopically express *CETS* family genes

**Figure 1:**
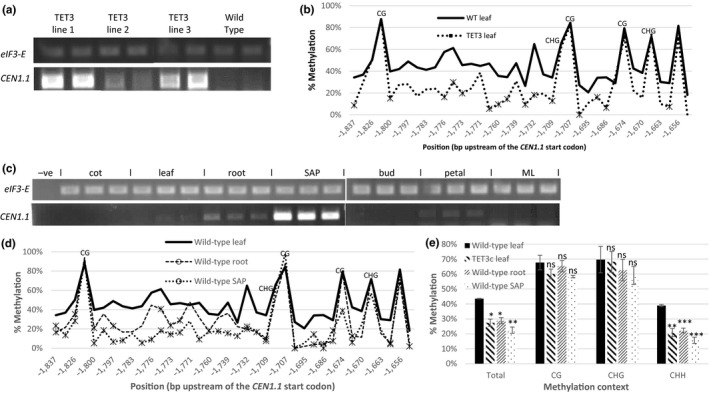
*CEN1.1* expression correlates with reduced methylation at CHH sites in *TET3c* and wild‐type tomato. (a) Ectopic expression of *CEN1.1* can be seen in the leaves of different lines of *TET3c* tomato plants (5 weeks old) using semiquantitative RT‐PCR on cDNA pools (*n* > 5). cDNA levels were normalized using the constitutively expressed eukaryotic translation initiation factor 3 subunit E. Two replicates are shown for each pool. (b) Reduced DNA methylation levels upstream of the *CEN1.1* gene were seen in *TET3c* leaves in comparison with wild‐type leaves. A 206‐bp region was analyzed by bisulfite sequencing. Three biological replicates were averaged for each genotype. CG and CHG sites are indicated; all unlabeled sites are CHH sites. Points where methylation is significantly different are marked with a star (*p* < .05, calculated using Student's two‐tailed *t* test). (c) *CEN1.1* is expressed in the shoot apex of wild‐type tomato, as well as roots and petals. Semiquantitative RT‐PCR was used to analyze expression of *CEN1.1* in a collection of cDNA pools (*n* > 3) from different wild‐type tomato tissues. cDNA levels were normalized using the constitutively expressed eukaryotic translation initiation factor 3 subunit E. The negative control (−ve) corresponds to a lane where DNA was not added. Cot = cotyledon, leaf = true leaves of 5‐week‐old tomato, SAP = shoot apices, ML = leaves of a mature (20 weeks old) tomato. (d) Reduced methylation levels were seen in root and SAP DNA where *CEN1.1* is expressed using the same 206‐bp region previously analyzed in *TET3c* tomato. Bisulfite sequencing was used to analyze methylation levels in 5‐week‐old leaf, root, and shoot apex from wild‐type tomato. Three biological replicates were averaged for each tissue. CG and CHG sites are indicated; all unlabeled sites are CHH sites. Points where methylation is significantly different are marked with a star (*p* < .05, calculated using Student's two‐tailed *t* test). (e) Methylation levels were reduced in the CHH context in *TET3c* leaves, wild‐type roots and wild‐type SAP. Graphs show averages with error bars representing standard error. *p < .05, **p < .005, ***p < .0005, ns = not significant, calculated using Student's two‐tailed *t* test

The *TET3c* construct, which consists of the catalytic domain of the mammalian DNA demethylase *TET3* under a constitutive *35S* promoter, has previously been shown to induce DNA hypomethylation in Arabidopsis (Hollwey et al., 2016)*. TET3c* was transformed into tomato plants in order to identify genes and processes affected by DNA methylation in tomato. Transgenic tomato plants, which strongly expressed *TET3c,* displayed a broad range of phenotypes, in particular an increase in primary shoot length and in the number of leaves in the primary shoot (Fig. S1a–d). These phenotypes have previously been observed in *35S::SP* plants (Shalit et al., 2009), and we therefore analyzed cDNA from *TET3c* tomato for changes in expression of *SP* and a selection of other genes from the *CETS/PEBP* gene family (Fig. S2a). Gene expression changes are seen in three genes, only two of which, *CEN1.1* and *SP9D,* showed a consistent increase in its expression in all *TET3c* lines in comparison with wild type. *CEN1.1 (Solyc03 g026050.2.1*) and *SP9D (Solyc09 g009560.1.1)* were not expressed in leaves from 5‐week‐old wild‐type tomato plants, but were expressed in leaves from 5‐week‐old *TET3c* tomato transformants (Figure 1a, Fig. S2a).

### 3.2 *TET3c* causes demethylation upstream of the *CEN1.1* gene

We screened the tomato database (Zhong et al., 2013) for methylation patterns of the known *CETS* gene family members and found that several members of the *CETS* gene family are methylated in the first 3 kb upstream of the transcriptional start site. With 37% total methylation, *CEN1.1* shows the highest levels of DNA methylation of all *CETS* genes analyzed. This includes 31% methylation of cytosines in a CHH (H=C, T or A) context, the context which shows the lowest methylation levels in plants. In comparison, *SP9D* had low levels of methylation upstream of the transcriptional start site, and therefore, further analysis was focused on *CEN1.1*. To investigate methylation levels upstream of *CEN1.1*, bisulfite sequencing was performed on DNA from leaf tissue of wild‐type and *TET3c* plants. A 200‐bp region with dense methylation in the tomato methylation database and homology to the tomato RK01 TRIM retrotransposon was chosen for analysis. DNA from wild‐type leaf tissue where *CEN1.1* was not expressed showed methylation levels of at least 40% for most CHH sites in this region. In *TET3c* plants with ectopic expression of *CEN1.1*, methylation levels were reduced by at least 50% for most CHH sites (Figure 1b, Fig. S2b). To confirm that the reduction in 5mC levels was caused by *TET3c*, we screened the region for 5‐hydroxymethylcytosine, a derivative of 5‐methylcytosine produced by *TET3* oxidation, which serves as a marker for *TET3c*‐mediated demethylation (Ito et al., 2010). Oxidative bisulfite sequencing showed that a significant increase in levels of 5‐hydroxymethylcytosine occurred in *TET3c* tissue compared to wild‐type tissue (Fig. S2c).

### 3.3 Tissue‐specific expression of *CEN1.1* correlates with DNA methylation

*CEN1.1* is characterized as a TFL1‐like member of the *CETS/PEBP* family based on its DNA sequence (Cao et al., 2016; Chardon & Damerval, 2005). Overexpression of other TFL1‐like genes, including *SP* in tomato and *RCN1/2* in rice, causes a delay in flowering, as does *TFL1* itself (Nakagawa, Shimamoto, & Kyozuka, 2002; Pnueli et al., 1998; Ratcliffe et al., 1998), suggesting that *CEN1.1* may be the cause of the phenotype observed in the *35S::TET3c* plants.

We used semiquantitative RT‐PCR to analyze the expression patterns of *CEN1.1* in wild‐type tomato. *CEN1.1* was not expressed in plant leaves in both juvenile (5 weeks old) and mature (20 weeks old) tomato plants, but was expressed strongly in the shoot apex and also weakly in roots and petals (Figure 1c). Bisulfite sequencing was used to analyze whether expression of *CEN1.1* correlated with hypomethylation in wild‐type tissues as it does in TET3c plants. Methylation levels were reduced in root and shoot apex (SAP) tissue where *CEN1.1* is expressed, in comparison with leaf tissue where *CEN1.1* is silenced (Figure 1d, Fig. S2d). In *TET3c,* root and SAP tissues, hypomethylation was observed at CHH sites, while overall CG and CHG methylation did not change significantly (Figure 1e).

### 3.4 Ectopic expression of *CEN1.1* causes increased vegetative meristematic identity

As discussed earlier, *TET3c* tomato plants ectopically expressing *CEN1.1* displayed increased primary stem length and increased stem thickness. To determine if these phenotypes were being caused by *CEN1.1* expression, the *CEN1.1* gene was cloned behind the constitutive 35S promoter in a plant expression vector and transformed into tomato. Transformants were selected that expressed the *CEN1.1* transgene, and progeny plants were analyzed 18 weeks after germination.

**Figure 2:**
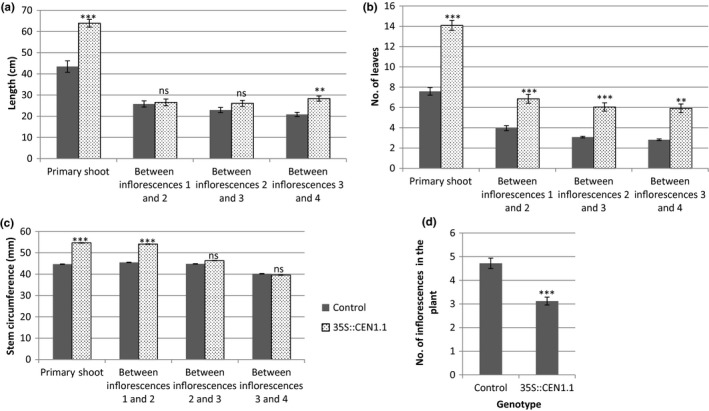
*35S::CEN1.1* plants have similar increased vegetative growth features as observed in *35S::TET3c* plants and also show a reduction in the number of sympodial units. (a, b) *35S::CEN1.1* plants (*n* = 34) have more leaves between each inflorescence than the control (*n* = 25), and also a greater primary shoot length. The number of leaves and distance between each inflorescence is shown separately, with the distance to the first inflorescence labeled as “Primary Shoot.” (c) Stem circumference is increased in *35S:CEN1.1* plants in comparison with tomato control plants. (d) *35S::CEN1.1* plants have a reduced number of inflorescences. Graphs show averages with error bars representing standard error. **p < .005, ***p < .0005, ns = not significant, calculated by Student's two‐tailed *t* test

**Figure 3:**
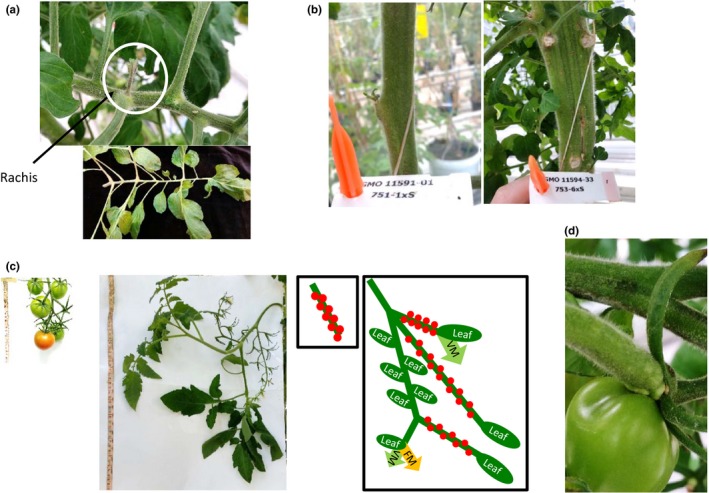
Increased vegetative growth in *35S::CEN1.1* plants results in abnormal phenotypes in a number of tissues. (a) Meristems grow directly out of the rachis of *35S::CEN1.1* tomato leaves (above), indicated by the white circle. (b) *35S::CEN1.1* plants (right) frequently show stem fasciation in older stems, not present in the control (left), resulting in increased stem circumference. (c) Images and diagrams comparing normal inflorescence growth and the leafy inflorescences of *35S::CEN1.1* plants. Normal growth of a tomato inflorescence is shown on the left. A leafy inflorescence from a plant ectopically expressing *CEN1.1* is shown on the right. Both inflorescences are the second inflorescence on the plant. The measuring tape is included as a size marker. VM = growth of a vegetative meristem, FM = growth of an inflorescence meristem. (d) Ectopic vegetative meristems emerging from the fruit of a *35S::CEN1.1* tomato

*35S::CEN1.1* tomato plants displayed an increased propensity for vegetative growth in comparison with control plants, as had the *TET3c* plants. Increases in primary shoot length, the number of leaves between inflorescences, and stem circumference were again observed (Figure 2a‐c). Despite this increase in the number of leaves between inflorescences, *35S::CEN1.1* plants were smaller overall than control plants, due to a significant reduction in the number of inflorescences present in the plant at 18 weeks (Figure 2d), a phenotype which had not been observed in *TET3c* plants. The increased level of vegetative growth could also be seen elsewhere. Vegetative meristems grew from the rachis of complex leaves in 73% of *35S::CEN1.1* plants (*n* = 33), but not in the tomato control plants (*n* = 25; Figure 3a). Stems of *35S::CEN1.1* plants were frequently fasciated (Figure 3b), a possible cause of their increased circumference (Figure 2c). In the inflorescence, we observed unusual vegetative growth. Leafy inflorescences on the *35S::CEN1.1* plants produced fruits and then switched back to a vegetative state for a time, before returning to an inflorescent state (Figure 3c). This pattern was reiterated on multiple branches that emerged in the inflorescences, suggesting that neither floral nor vegetative identity could be stably maintained. The reversion to vegetative meristematic growth even continued in a small number of tomato fruit, with a vegetative meristem growing from the top of the fruit in 0.8% of fruit (*n* = 352; Figure 3d).

### 3.5 *CEN1.1* leafy inflorescences switch between the inflorescence and vegetative state resulting in an increased number of flowers

**Figure 4:**
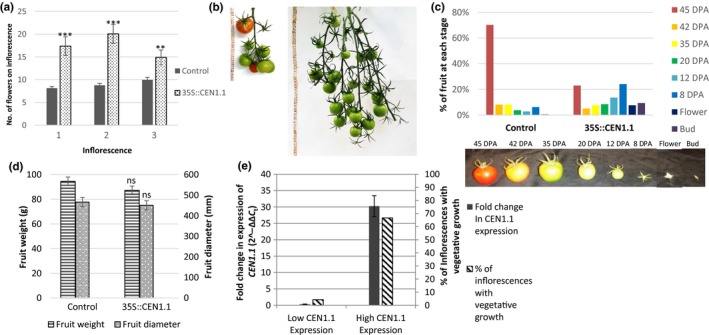
Inflorescences of *35S::CEN1.1* plants produced an increased number of flowers but were delayed in development. (a) Inflorescences of *CEN1.1* (*n* = 34) produced an increased number of flowers compared to the control (*n* = 28). (b) An inflorescence from a *35S::CEN1.1* plant from which all leaves and vegetative meristems were removed as the inflorescence developed and a control inflorescence with a normal number of fruit. Both are the first inflorescence on the plant. (c) Fruits on *35S::CEN1.1* plants (*n* = 208) were less developed than fruits on the equivalent inflorescence of a control plant (*n* = 352). Fruits on the first inflorescence were categorized according to ripeness, or recorded as flowers. DPA—days postanthesis. (d) Ripe fruits were similar in size and weight on control and *35S::CEN1.1* plants. The widest diameter of each tomato fruit was used as a measurement; 11 plants were measured each for the control and *CEN1.1*. (e) Ectopic expression of *CEN1.1* correlates with the leafy inflorescence phenotype. Plants categorized as “High *CEN1.1* Expression” (greater than fivefold increase in *CEN1.1* expression in comparison with the control, *n* = 18) were compared to plants categorized as “Low *CEN1.1* Expression” *(CEN1.1* expression did not increase in comparison with the control, *n* = 3). Graphs show averages with error bars representing standard error. *p < .05, **p < .005, ***p < .0005, ns = not significant, calculated by Student's two‐tailed *t* test

Large numbers of inflorescences of *35S::CEN1.1* plants (76%, *n* = 76) were leafy in comparison with control plants (0%, *n* = 17), a phenotype which had also been seen in 18% of *TET3c* inflorescences (*n* = 51; Fig. S1d). Inflorescences were classified as leafy when they contained multiple leaves and at least one vegetative meristem. Inflorescences containing leaves have also been described for lines that overexpress *SP*, although the reported effects are less severe than the ones we observed (Pnueli et al., 1998), and in *sft, macrocalyx,* or *jointless* mutants (Quinet, 2006; Vrebalov et al., 2002). Expression of these genes remains unchanged in the *35S::CEN1.1* tomato, which argues against *CEN1.1* overexpression altering their expression (Fig. S3). While these abnormal, leafy inflorescences contained large quantities of vegetative material, they also produced a larger number of flowers due to the large branched nature of the inflorescence. Therefore, *35S::CEN1.1* inflorescences also produce more flowers on average than wild‐type inflorescences (Figure 4a), although the number of flowers on an inflorescence varied significantly, ranging from 11 to 60. This phenotype becomes more obvious when vegetative material is removed during the development of the inflorescence (Figure 4b). While it required more time for *35S::CEN1.1* lines to produce fully ripe flowers (Figure 4c), there was no significant difference in fruit size or weight (Figure 4d). Mutants of *COMPOUND INFLORESCENCE (S)* or *ANANTHA (AN)* can also produce branched inflorescences with an increased number of flowers (Lippman et al., 2008), but expression of these genes was unchanged in the *CEN1.1* tomato (Fig. S3). Among the plants that we had selected on the basis that they contained the *35S::CEN1.1* construct, we identified three plants that no longer expressed the transgene. Silencing of transgenes after successful transformation can subsequently become silenced in plants for many reasons (Meyer & Heidmann, 1994). All three plants resembled the wild‐type phenotype (Figure 4e, Fig. S4), providing further support that the observed phenotypes result from ectopic *CEN1.1* expression.

### 3.6 *CEN1.1* expression in *Arabidopsis thaliana* delays or prevents flowering

**Figure 5:**
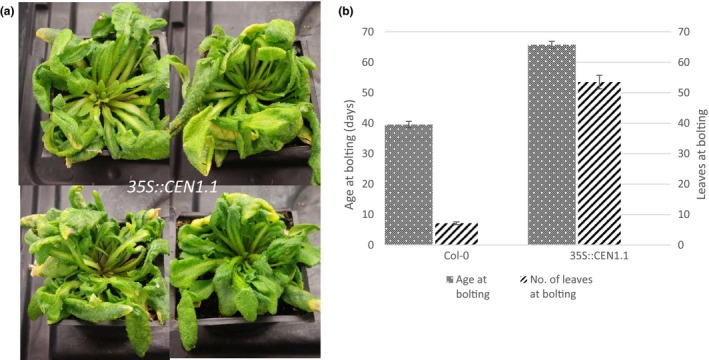
*Arabidopsis thaliana* transformed with *35S::CEN1.1* showed an increase in vegetative growth and flowering was delayed or absent. (a) Arabidopsis plants containing *35S::CEN1.1* had not flowered after 9 weeks but continued to produce rosette leaves. (b) 50% of *35S::CEN1.1* Arabidopsis (*n* = 8) were able to flower. Those that did flower, flowered late and produced a higher number of rosette leaves prior to bolting (*n* = 4) in comparison with Col‐0 (*n* = 12). Graphs show averages with error bars representing standard error

To further verify the action of *CEN1.1* as a floral repressor, the *35S::CEN1.1* construct was transferred into *Arabidopsis thaliana*. Eight independent transformant lines were grown under long day conditions. Four plants failed to flower completely, dying after 12 weeks without flowering. The other four plants did flower at late stages. While *Col‐0* wild type flowered on average 39 days after germination when seven rosette leaves had been produced, *35S::CEN1.1* transformants flowered on average 65 days after germination when 53 rosette leaves had been produced (Figure 5).

## 4 DISCUSSION

### 4.1 *CEN1.1* is the first tomato *CETS* gene with a demonstrated link to DNA methylation

Thirteen members of the *CETS/PEBP* gene family are characterized in tomato, and five of these genes have been shown to act in the control of shoot architecture and timing of the floral transition (Cao et al., 2016; Shalit et al., 2009). Our analysis of *CEN1.1* demonstrates that it also plays a role in this process and that it is the first of these genes to be shown to be affected by DNA methylation. The normal biological function of *CEN1.1* is unknown, but its expression pattern (strong expression only in the shoot apex) suggests that it may act in the regulation of shoot architecture. Intensity of activation of *CEN1.1* expression correlates with increasing hypomethylation in its promoter, suggesting that the expression of *CEN1.1* is connected to DNA methylation of CHH sites in the promoter. The CHH methylation levels upstream of the *CEN1.1* gene are unusually high, compared to an overall level of 8.6% in the tomato genome (Zhong et al., 2013). High levels of methylation in all three contexts is known as dense methylation, which has been shown to be dependent on the *MET1* gene in some genes in Arabidopsis (Watson, Hawkes, & Meyer, 2014), but it is unknown if this is the case in tomato.

### 4.2 *CEN1.1* acts as a floral repressor in tomato and Arabidopsis

Unsurprisingly, given its similarity and close phylogenetic relationship to *SP*, ectopic expression of *CEN1.1* has similar effects to ectopic expression of *SP*. Expression of both genes causes delayed termination of the primary shoot, with an increase in the number of leaves in the primary shoot and in subsequent sympodial units. In Arabidopsis, expression of *CEN1.1* either delayed or prevented flowering. Similarly, strong effects of *CETS* genes have been reported in other species; for example, the expression of the Antirrhinum floral repressor gene *CEN* in tobacco resulted in significant delays in flowering, with some plants being delayed for over 10 months, and one never flowering at all (Amaya, Ratcliffe, & Bradley, 1999). The delay in flowering caused by *CEN1.1* in Arabidopsis was more severe than was observed when the Arabidopsis *CEN1.1* homologues, *TFL1* and *BFT*, were expressed under the 35S promoter (Mimida et al., 2001; Yoo et al., 2010). As would be expected, ectopic expression of the tomato *SFT* gene in Arabidopsis has the opposite effect to *CEN1.1*, causing early flowering after the production of four rosette leaves (Cao et al., 2016).

*CEN1.1* may bind the same targets as *SP*, resulting in activation of the same pathway or may act through a different route. Like the rest of the *CETS* gene family, *CEN1.1* possesses a PEBP domain, but the role of this domain in the function of the gene family has not yet been clarified. *SP* has been shown to interact with several proteins in tomato including a kinase, 14‐3‐3 proteins and a putative bZIP transcription factor (Pnueli et al., 2001; as does *FT* in Arabidopsis (Abe et al., 2005)), but the full pathway has not been elucidated and it is not known how the other known floral repressors in the tomato *CETS* gene family exert their influence (Cao et al., 2016). The development of leaves on inflorescences induced by ectopic expression of *CEN1.1* had also been observed in *sft, macrocalyx*,* and jointless* mutants (Quinet, 2006; Vrebalov et al., 2002). There was no indication that any of these genes altered their expression in *CEN1.1* transformants, which argues against their involvement in the phenotype observed in the transformants.

### 4.3 Increased vegetative growth caused by *CEN1.1* appears differently in various tissues and paradoxically increases total fruit yield

Ectopic expression of *CEN1.1* also stimulates vegetative growth elsewhere in tomato plants, which seems to be more severe than similar phenotypes described in *35S::SP* tomato plants (Quinet, 2006). This presents differently in different plant tissues. In leaves, ectopic expression results in the presence of vegetative meristems emerging from the leaf. In stems, ectopic expression results in fasciation of the stem, and thus thicker stems. Inflorescences with ectopic expression of *35S::CEN1.1* are unable to finally commit to the inflorescent state, but switch repeatedly between a vegetative and an inflorescent state. This results in a greater quantity of fruit from a single tomato inflorescence, which could be advantageous. The average *35S::CEN1.1* inflorescence produces 22 flowers, in comparison with the 10 produced by a control inflorescence. Despite the reduced number of inflorescences per plant (3.1 c.f. 4.7), this still results in an increased yield of fruit from a single tomato plant, with an average of 68 fruits per *35S::CEN1.1* plant and only 47 fruits per control plant. Fruits from *35S::CEN1.1* plants, once ripe, have the same size as fruits from control plants, but more time is required for the fruits to become fully ripe. The median fruit on the first inflorescence of an 18‐week‐old control plant is ready to be removed (45 days postanthesis (D.P.A), while the median fruit on the first inflorescence of a *35S::CEN1.1* plant is still small and green (12 D.P.A.) on an 18‐week‐old plant. *35S::CEN1.1* plants would therefore require an extra 5 weeks for fruit to fully ripen, or 26% of the total growth time.

*35S::CEN1.1* plants produce 45% more fruit than the control, although for them to ripen takes 26% longer. The reduced number of inflorescences per plant also means that *35S::CEN1.1* tomato plants are smaller despite the increase in the number of leaves between inflorescences, and therefore, more tomatoes can be produced in a smaller glasshouse space. However, pruning will be required to prevent effects on the harvest index (total yield per plant weight) due to the vegetative growth on the inflorescence.

### 4.4 *CEN1.1* was identified using *TET3c,* which could be of use in identifying other methylation‐linked genes in tomato and other species

Expression of the mammalian demethylase *TET3c* in tomato facilitated the identification of the *CEN1.1* gene. Phenotypes seen in *35S::CEN1.1* plants had already been observed in *TET3c* plants, although often at a lower frequency or intensity. This is to be expected, given that *CEN1.1* expression due to *TET3c‐*mediated demethylation is likely to be less intense than the strong, constitutive expression under the 35S promoter.

Identification of the *CEN1.1* gene illustrates that *TET3c* expression is a useful tool to discover previously unknown plant genes that are affected by DNA methylation changes. These may be otherwise difficult to detect, especially in species which are particularly susceptible to changes in DNA methylation. Arabidopsis mutants of the main methyltransferases are still viable. This allows high‐throughput analysis of changes in DNA methylation and gene expression, which can identify genes controlled by methylation. In contrast, species such as tomato and rice appear to be more sensitive to DNA methylation changes as they show more adverse effects when the enzymes involved in DNA methylation are lost (Liu et al., 2015; Ono et al., 2012). In tomato, null mutations of *SlNRPE1*, a component of the RdDM pathway, are lethal (Gouil & Baulcombe, 2016), and *MET1* RNAi lines are not viable (Watson, 2013), making the identification of genes and processes affected by DNA methylation changes more challenging. *TET3c* expression may therefore offer an alternative in these species to gain a better understanding of which processes are controlled by DNA methylation.

## AUTHOR CONTRIBUTIONS

P.M. and I.H. supervised the overall study, which was conducted largely by E.H with input from M.R.W. Tomato transformation and plant cultivation were carried out by M.R.W. and S.O. E.H. wrote the article with input from P.M. and I.H. All authors read and approved the final manuscript.
