# Supplementary figures and images for: TET3‐mediated demethylation in tomato activates expression of a CETS gene that stimulates vegetative growth
Source: Plant Direct. 2017 Oct 30;1(4):e00022. doi: 10.1002/pld3.22 (PMC6508569; doi:10.1002/pld3.22)

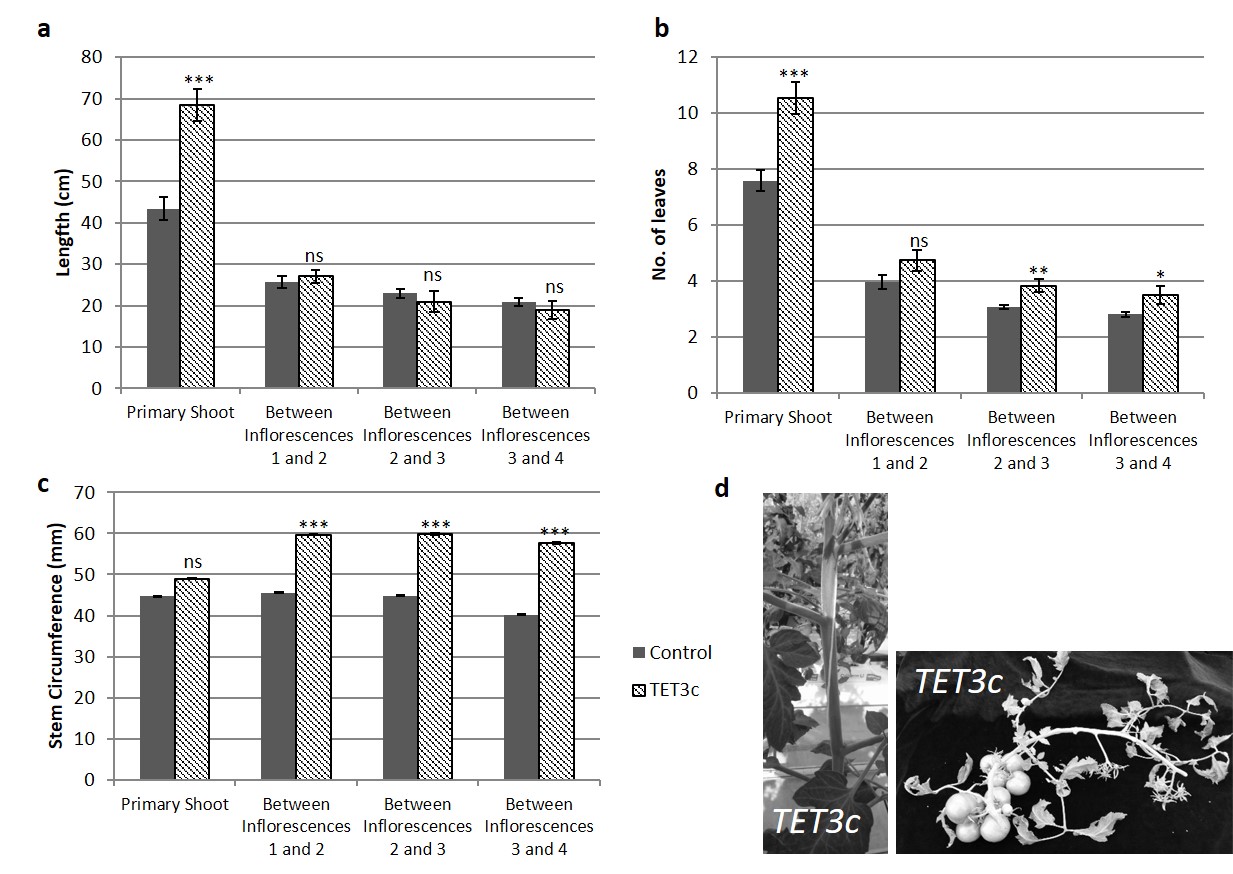

Supplement: Supplementary file 1 [file PLD3-1-e00022-s001.jpg]

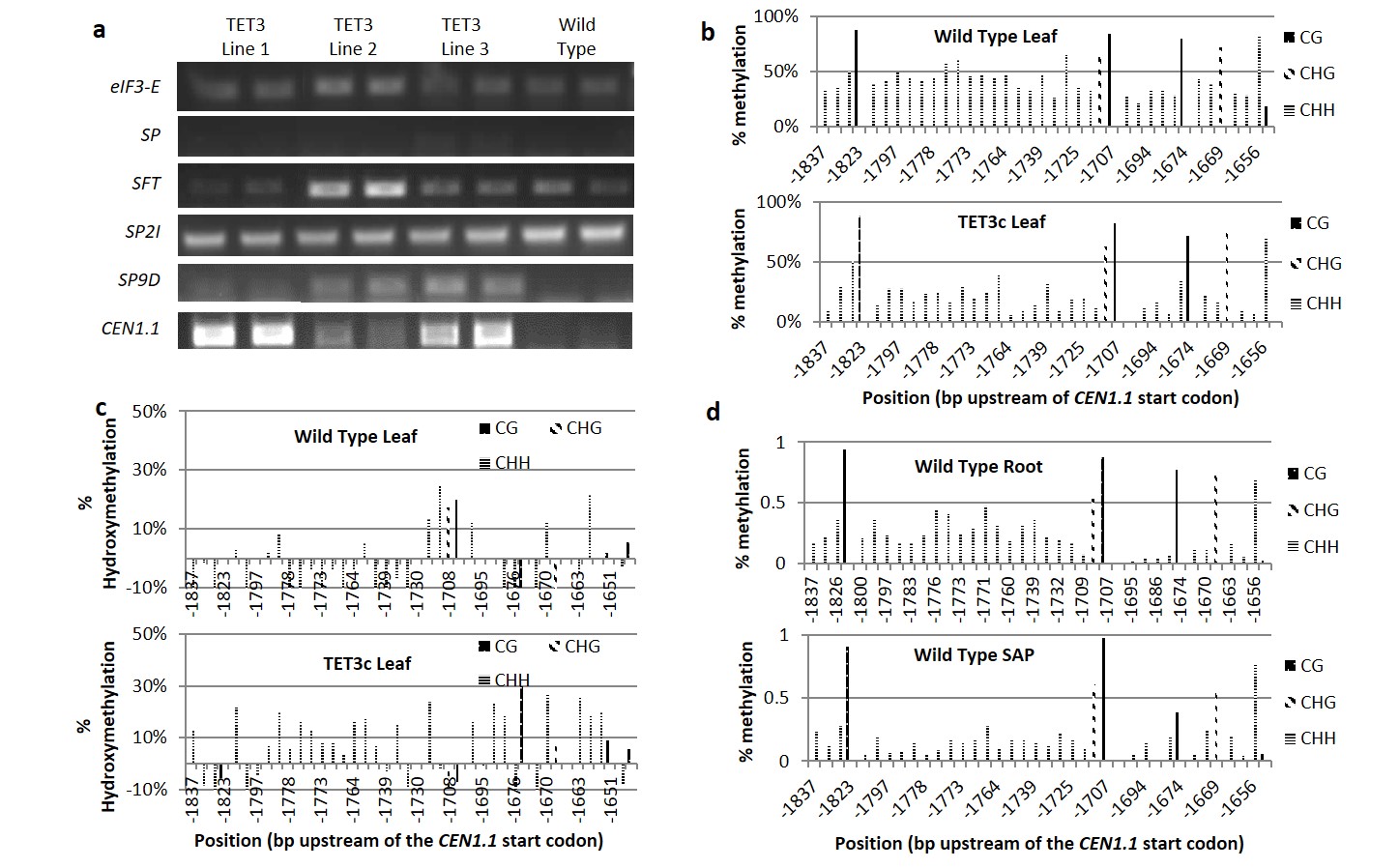

Supplement: Supplementary file 2 [file PLD3-1-e00022-s002.jpg]

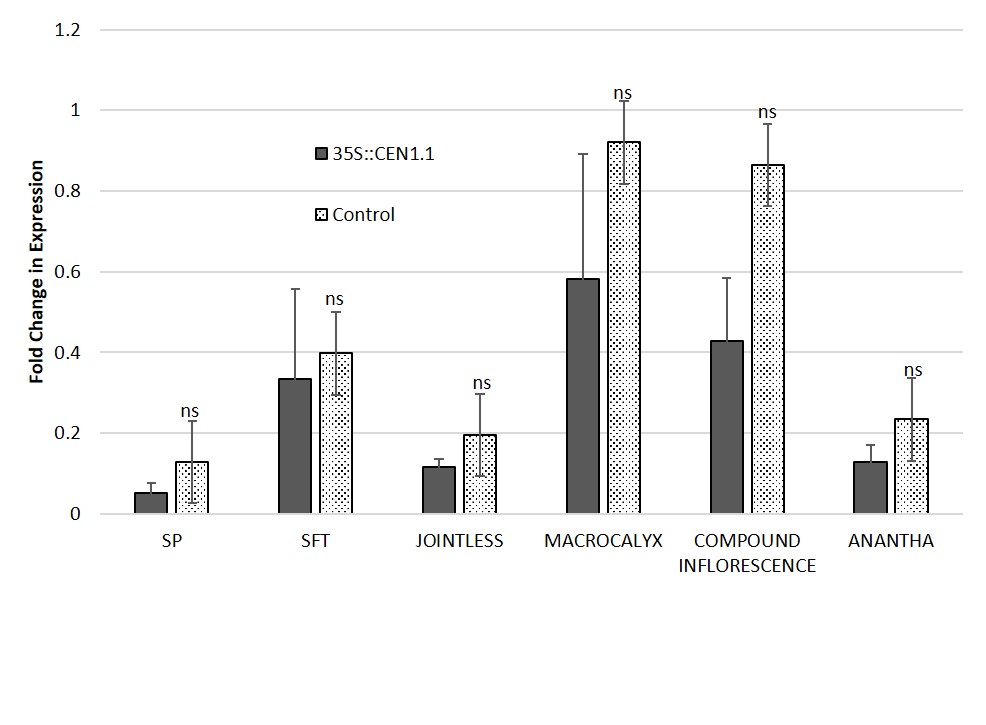

Supplement: Supplementary file 3 [file PLD3-1-e00022-s003.jpg]

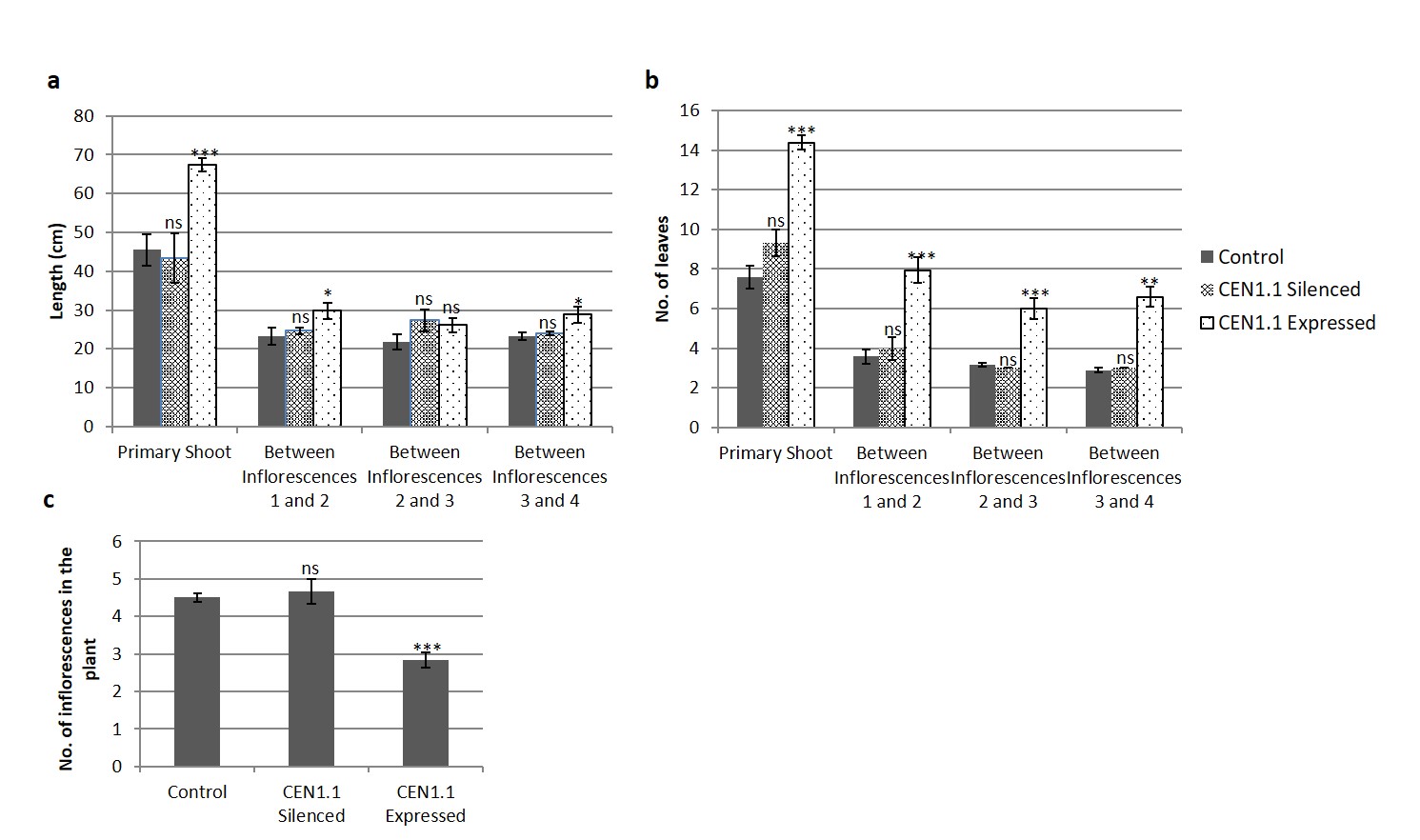

Supplement: Supplementary file 4 [file PLD3-1-e00022-s004.jpg]
